# Association of lower serum and follicular fluid vitamin D levels with reduced fertilization rates in IVF patients with diminished ovarian reserve

**DOI:** 10.3389/fendo.2026.1750481

**Published:** 2026-04-10

**Authors:** Aiping Zhang, Yanjun Shi, Yuzi Li, Xiaolong Li, Feifei Xu, Qianqian Hong, Feng Yue, Bin Wang, Haofei Shen, Xuehong Zhang

**Affiliations:** 1 The First Clinical Medical College, Lanzhou University, Lanzhou, Gansu, China; 2 The First Hospital of Lanzhou University, Lanzhou, Gansu, China; 3 Key Laboratory for Reproductive Medicine and Embryo Gansu Province, Lanzhou, Gansu, China; 4 School of Public Health, Lanzhou University, Lanzhou, Gansu, China; 5 Department of Laboratory Medicine, The First Hospital of Lanzhou University, Lanzhou, Gansu, China; 6 Clinical Medicine, First Clinical Medical College, Gansu University of Traditional Chinese Medicine, Lanzhou, Gansu, China

**Keywords:** diminished ovarian reserve, vitamin D, serum, follicular fluid, in vitro fertilization

## Abstract

**Introduction:**

This study aimed to investigate the association between 25-hydroxyvitamin D [25(OH)D] levels in serum (VD-S) and follicular fluid (VD-FF) and the laboratory outcomes of *in vitro* fertilization (IVF) in patients with diminished ovarian reserve (DOR).

**Methods:**

This prospective cohort study enrolled 145 women undergoing IVF, comprising 74 patients with DOR and 71 with normal ovarian reserve (NOR) as controls. VD-S and VD-FF levels were measured. The DOR group was further stratified into vitamin D (VD) non-deficient (VDH) and VD deficient (VDL) subgroups. Baseline characteristics and IVF laboratory outcomes were compared between the groups and subgroups.

**Results:**

A significant positive correlation was observed between VD-S and VD-FF levels (r = 0.769, *P* < 0.001). Patients in the DOR group had significantly lower 25(OH)D levels in both serum (*P* = 0.048) and follicular fluid (*P* = 0.015) compared to the NOR group. Within the DOR cohort, the VDH subgroup demonstrated significantly higher oocyte retrieval rates (95.0% *vs*. 84.6%, *P* = 0.021) and normal fertilization rates (72.9% *vs*. 56.3%, *P* = 0.016) than the VDL subgroup when stratified by VD-S. Similar results were found when stratifying by follicular fluid levels. Furthermore, the VDH subgroup exhibited significantly higher high-density lipoprotein (HDL) levels (*P* < 0.05).

**Conclusion:**

In patients with diminished ovarian reserve, higher 25(OH)D levels in serum and follicular fluid are positively associated with higher oocyte retrieval and normal fertilization rates during IVF cycles. These findings suggest that VD deficiency may be a modifiable factor associated with IVF outcomes in this specific population.

## 1 Introduction

Ovarian reserve pertains to the size and quality of the remaining primordial follicle pool and is clinically reflected by the number of recruitable antral follicles. It is susceptible to numerous factors, including initial ovarian reserve, age, hormones, diseases, medications, metabolites, and environmental concerns (1, 2).Diminished ovarian reserve (DOR) generally denotes a decline in the quantity and/or quality of oocytes resulting from various causes, ultimately leading to insufficient ovarian function (3). While assisted reproductive technology (ART) has emerged as the primary approach to address the fertility issues of women with DOR, its success rate remains constrained, largely due to poor oocyte quality (4, 5). This limitation presents a significant clinical challenge and places a heavy burden on patients. Consequently, there is a pressing clinical need to identify modifiable factors beyond current treatment protocols that could enhance oocyte quality and thereby improve ART outcomes, representing a critical research frontier in reproductive medicine.

Vitamin D (VD), an indispensable fat-soluble steroid hormone, orchestrates multiple signaling pathways in the human body, including those in bone and calcium homeostasis, inflammation, and immunity (6, 7). VD is synthesized in the skin upon ultraviolet exposure or obtained through diet, subsequently undergoing hydroxylation in the liver to form 25-hydroxyvitamin D [25(OH)D] (8). Although 1,25-dihydroxyvitamin D [1,25(OH)_2_D] is the biologically active form, 25(OH)D is the preferred clinical indicator for assessing vitamin D status due to its longer half-life and superior stability, providing a more reliable reflection of total body vitamin D stores (9). A growing body of evidence has demonstrated that VD also exerts regulatory effects on the female reproductive system (10). Research suggests that severe systemic VD deficiency may be linked to a reduction in ovarian reserve (11, 12). Furthermore, VD supplementation in patients with both DOR and VD deficiency has been shown to decrease follicle-stimulating hormone (FSH) levels, suggesting it could be a potential therapeutic option to improve fertility outcomes in this population (13).

However, the findings from studies investigating the influence of endogenous VD levels on reproductive outcomes have been inconsistent and remain a topic of significant debate (14, 15). Moreover, research has predominantly centered on serum 25(OH)D levels (VD-S) (14). There remains a scarcity of studies that specifically focus on the DOR population while simultaneously exploring the relationship between VD levels in the follicular microenvironment (follicular fluid) and *in vitro* fertilization (IVF) outcomes.

Therefore, this study was designed to specifically address this gap by investigating the correlation between 25(OH)D levels in both serum and follicular fluid and the laboratory outcomes of IVF among patients with DOR. The findings aim to provide a theoretical foundation for optimizing assisted reproductive strategies for this specific patient population.

## 2 Materials and methods

### 2.1 Study design

This prospective cohort study was conducted at the Reproductive Medicine Center of the First Hospital of Lanzhou University between November 2023 and January 2024. This period represents the time of minimum ultraviolet B (UVB) radiation in Lanzhou, China, ensuring that the measured 25(OH)D levels accurately reflect the patients’ baseline status and the prevalence of vitamin D deficiency in this population. The study protocol was approved by the Reproductive Medicine Ethics Committee of the First Hospital of Lanzhou University (Approval No. LDYYSZLLKH2025-04). All participants provided written informed consent prior to inclusion.

### 2.2 Study participants

A total of 145 infertile women were enrolled and divided into two groups, DOR Group (n=74): Patients diagnosed with diminished ovarian reserve (DOR). NOR Group (n=71): Patients with normal ovarian function undergoing IVF for tubal factors, serving as the control group.

Based on serum 25(OH)D levels and in accordance with the “Expert Consensus on the Evaluation and Improvement of Vitamin D Nutritional Status in China (2023) (16)”, vitamin D status is classified as deficient (< 12 ng/mL [30 nmol/L]), insufficient (12 to < 20 ng/mL [30 to < 50 nmol/L]), and sufficient (≥ 20 ng/mL [50 nmol/L]). In this study, patients with insufficient and sufficient levels were combined into a “vitamin D non-deficient” subgroup (VDH, ≥ 12 ng/mL), while those with deficient levels were categorized into a “vitamin D deficient” subgroup (VDL, < 12 ng/mL).

Inclusion Criteria: (1) Age ≤ 40 years. (2) For DOR patients, the diagnosis of Diminished Ovarian Reserve (DOR) was established according to the Consensus on Clinical Diagnosis and Management of Diminished Ovarian Reserve (2022), which incorporates the thresholds for follicle reserve and AMH levels from the 2011 ESHRE Bologna criteria for poor ovarian response. Patients were diagnosed with DOR if they met at least two of the following three criteria (17, 18): ① Antral follicle count (AFC) < 5–7 follicles; ② Anti-Müllerian hormone (AMH) < 1.1 ng/ml; ③ Basal follicle-stimulating hormone (FSH) ≥ 10 IU/L. (3) Undergoing an IVF cycle with ovarian stimulation.

Exclusion Criteria: (1) Premature Ovarian Insufficiency (POI): To strictly distinguish from DOR, patients with POI were excluded based on the following criteria: age < 40 years, menstrual abnormalities (amenorrhea or oligomenorrhea for > 4 months), and FSH > 25 IU/L (measured twice with an interval of > 4 weeks) accompanied by fluctuating estradiol levels. (2) Other conditions including endometriosis (stage III - IV), polycystic ovary syndrome, premature ovarian insufficiency, uterine malformations, endometrial pathologies, chromosomal abnormalities, autoimmune disorders, chronic systemic diseases, and the male presents with moderate to severe oligoasthenospermia.

### 2.3 Sample collection

On the day of human chorionic gonadotropin (hCG) injection, serum samples were collected. These samples were centrifuged at 4000 x g for 10 minutes and then stored at -80 °C until the day of analysis. On the day of oocyte retrieval, the first aliquot of clear, mixed follicular fluid was collected under the guidance of transvaginal ultrasound. This fluid was centrifuged using the same protocol and stored at -80 °C until the day of testing.

### 2.4 Laboratory measurements and outcome variables

The primary variables, 25(OH)D levels in serum and follicular fluid, as well as baseline reproductive hormones (FSH, LH, E_2_, P_4_, PRL, AMH, and TSH), were quantified using a Roche Cobas e801 fully automated chemiluminescence immunoassay analyzer (Roche Diagnostics, Mannheim, Germany). All tests were performed using proprietary Roche Cobas reagent kits according to the manufacturer’s instructions. This method was selected as it is the standardized clinical detection tool provided to all patients at the First Hospital of Lanzhou University, ensuring that our findings are directly applicable to routine clinical practice. The within-batch and between-batch coefficients of variation (CV) were 5.5% and 10%, respectively.

Baseline Variables: On days 2–3 of the menstrual cycle, levels of follicle - stimulating hormone (FSH), luteinizing hormone (LH), estradiol (E_2_), progesterone (P_4_), prolactin (PRL), anti - Müllerian hormone (AMH), thyroid - stimulating hormone (TSH), and the basal antral follicle count (bAFC) were measured. Specifically, bAFC was assessed via transvaginal ultrasound (TVUS) using a 5–9 MHz probe on days 2–3 of the menstrual cycle. It was defined as the total number of follicles measuring 2–10 mm in diameter in both ovaries, with all examinations performed by the same experienced clinician to ensure consistency. Additionally, serum levels of homocysteine (HCY), fasting blood glucose (FBG), triglycerides (TG), total cholesterol (TC), high - density lipoprotein cholesterol (HDL), low - density lipoprotein cholesterol (LDL), and erythrocyte sedimentation rate (ESR) were determined.

Primary Measured Variables: VD-S on the trigger day and 25(OH)D levels in follicular fluid were the primary measured variables. Laboratory Variables during the IVF Cycle:

Variables including the oocyte retrieval rate (number of retrieved oocytes/number of punctured follicles × 100%), the metaphase II (MII) oocytes rate (number of MII oocytes/total oocytes retrieved × 100%), the normal fertilization rate (the number of oocytes presenting 2 pronuclei and 2 polar bodies on the first day of IVF/the total number of oocytes inseminated during IVF × 100%), the proportion of available embryos on day 3(number of usable embryos on the third day of IVF/number of normally fertilized oocytes × 100%), and the proportion of high-quality embryos on day 3 (number of high-quality embryos on the third day of IVF/number of normally fertilized oocytes × 100%) were recorded.

### 2.5 Statistical analyses

Measurement data were first tested for normality using the Shapiro - Wilk test. Since the data were found to be skewed, they were described using the median (M) and the interquartile range [P_25_, P_75_]. The non-parametric Mann-Whitney U test was employed to compare differences between groups. Subsequently, subgroup analyses were conducted based on clinical 25(OH)D thresholds (e.g., ≥12 ng/mL *vs*. <12 ng/mL), which were performed independently for both serum and follicular fluid samples. Categorical data were presented as percentages (%), and group comparisons were performed using the Pearson chi - square (χ²) test. The Spearman rank - correlation analysis was used to assess the relationships between variables. All statistical tests were two - tailed. A significance level of *P* < 0.05 was set to indicate statistically significant differences. Subgroup comparisons were carried out using the same statistical approaches described above. No adjustments for multiple comparisons were applied, as the subgroup analyses were considered exploratory and hypothesis-generating. The data analysis was conducted using SPSS 25.0 software.

## 3 Results

### 3.1 Baseline characteristics and IVF outcomes of the study population

A total of 145 subjects were enrolled, comprising 74 in the DOR group and 71 in the NOR group. No statistically significant differences were observed between the two groups in terms of age, type of infertility, duration of infertility, or body mass index (BMI) (*P* > 0.05). As expected, the DOR group exhibited significantly higher basal FSH levels (10.34 mIU/ml *vs*. 7.00 mIU/ml, *P* < 0.001) and significantly lower AMH levels (0.48 ng/mL *vs*. 2.39 ng/mL, P < 0.001) and antral follicle counts (AFC) (4 *vs*. 12, *P* < 0.001) compared to the NOR group. No significant differences were detected between the groups for basal LH, E_2_, P_4_, PRL, TSH, or for metabolic markers such as HCY, FBG, TG, TC, HDL, LDL, and ESR (*P* > 0.05).

Regarding VD levels, both VD-S and follicular fluid 25(OH)D (VD-FF) were significantly lower in the DOR group than in the NOR group (*P* = 0.048 and *P* = 0.015, respectively). In terms of IVF laboratory outcomes, the oocyte retrieval rate was significantly lower in the DOR group (88.8% *vs*. 92.5%, *P* = 0.042). However, no significant differences were found in the rates of mature oocytes, normal fertilization, available embryos on day 3, or high-quality embryos on day 3 (*P* > 0.05) (**Table 1**).

**Table 1:** The baseline characteristics of the patients, VD levels and *in vitro* fertilization parameters.

Index	DOR (n=74)	NOR (n=71)	*Z/χ²*	P
Age (year)	35.5[33.8-38.0]	36[31.0-38.0]	-0.731	0.465
Type of infertility, No. (%)				
Primary	26(35.1%)	29(40.8%)	0.502	0.479
Secondary	48(64.9%)	42(59.2%)		
Duration of infertility (years)	3.0 [1.0-6.0]	3.0 [2.0-5.0]	-0.555	0.579
BMI	22.35[20.78-25.25]	22.4[20.9-25.30]	-0.257	0.797
FSH (mIU/ml)	10.34[7.68-14.88]	7[5.89-8.00]	-6.071	<0.001
LH (mIU/ml)	5.70[3.32-8.64]	5.49[4.00-7.16]	-0.597	0.550
E2 (pg/ml)	42.45[20.43-61.33]	38.3[29.50-50.10]	-0.158	0.874
P4 (ng/mL)	0.26[0.17-0.36]	0.26[0.17-0.42]	-0.580	0.562
PRL (ng/mL)	18.45[14.43-26.45]	22.2[16.2-29.09]	-1.665	0.098
AMH (ng/mL)	0.48[0.24-0.71]	2.39[1.47-3.30]	-9.936	<0.001
TSH (mIU/ml)	2.78[1.80-4.51]	2.59[1.95-3.39]	-0.874	0.382
AFC	4[3-6]	12[9-16]	-9.476	<0.001
HCY((μmol/L)	10.65[8.75-15.00]	10.8[9.00-16.00]	-0.661	0.509
FBG (mmol/L)	4.75[4.50-5.21]	4.74[4.47-5.17]	-0.358	0.720
TG (mmol/L)	1.25[0.88-1.79]	1.39[0.83-2.15]	-.0829	0.407
TC (mmol/L)	4.41[3.90-5.03]	4.49[3.88-5.18]	-0.237	0.812
HDL (mmol/L)	1.43[1.25-1.78]	1.44[1.17-1.69]	-0.599	0.549
LDL (mmol/L)	2.70[2.43-3.24]	2.98[2.50-3.47]	-1.442	0.149
ESR(mm/h)	6[3-8]	7[5-10]	-1.664	0.096
VD-S (ng/mL)	8.90[5.60-13.38]	10.02[6.10-27.40]	-1.976	0.048
VD-FF (ng/mL)	8.00[3.58-13.23]	10.90[6.50-18.30]	-2.429	0.015
Oocytes retrieval rate (%)	88.8%(221/250)	92.5%(809/875)	4.140	0.042
Metaphase II oocytes rate (%)	89.1%(197/221)	91.3%(739/809)	1.020	0.313
Normal fertilization rate (%)	63.5%(125/197)	69.8%(516/739)	2.926	0.087
Available embryos rate of D3 (%)	64.8%(81/125)	68.2%(352/516)	0.536	0.464
High quality embryos rate of D3 (%)	25.6%(32/125)	25.8%(133/516)	0.002	0.968

25(OH)D levels are presented in ng/mL. To convert to nmol/L, multiply the values by 2.5 (e.g., 12 ng/mL) = 30 nmol/L.

FSH, follicle-stimulating hormone; LH, luteinizing hormone; E_2_, estradiol; P_4_, progesterone; PRL, prolactin; AMH, anti-Mullerian hormone; TSH, thyroid-stimulating hormone; bAFC, basal antral follicle count; FBG, fasting blood glucose; HCY, homocysteine; TG, triglycerides; TC, cholesterol; HDL, high-density lipoprotein; LDL, low-density lipoprotein; ESR, erythrocyte sedimentation rate; VD-S, 25(OH)D level in serum; VD-FF, 25(OH)D level in follicular fluid.

### 3.2 Correlation between serum and VD-FF Levels

**Figure 1:**
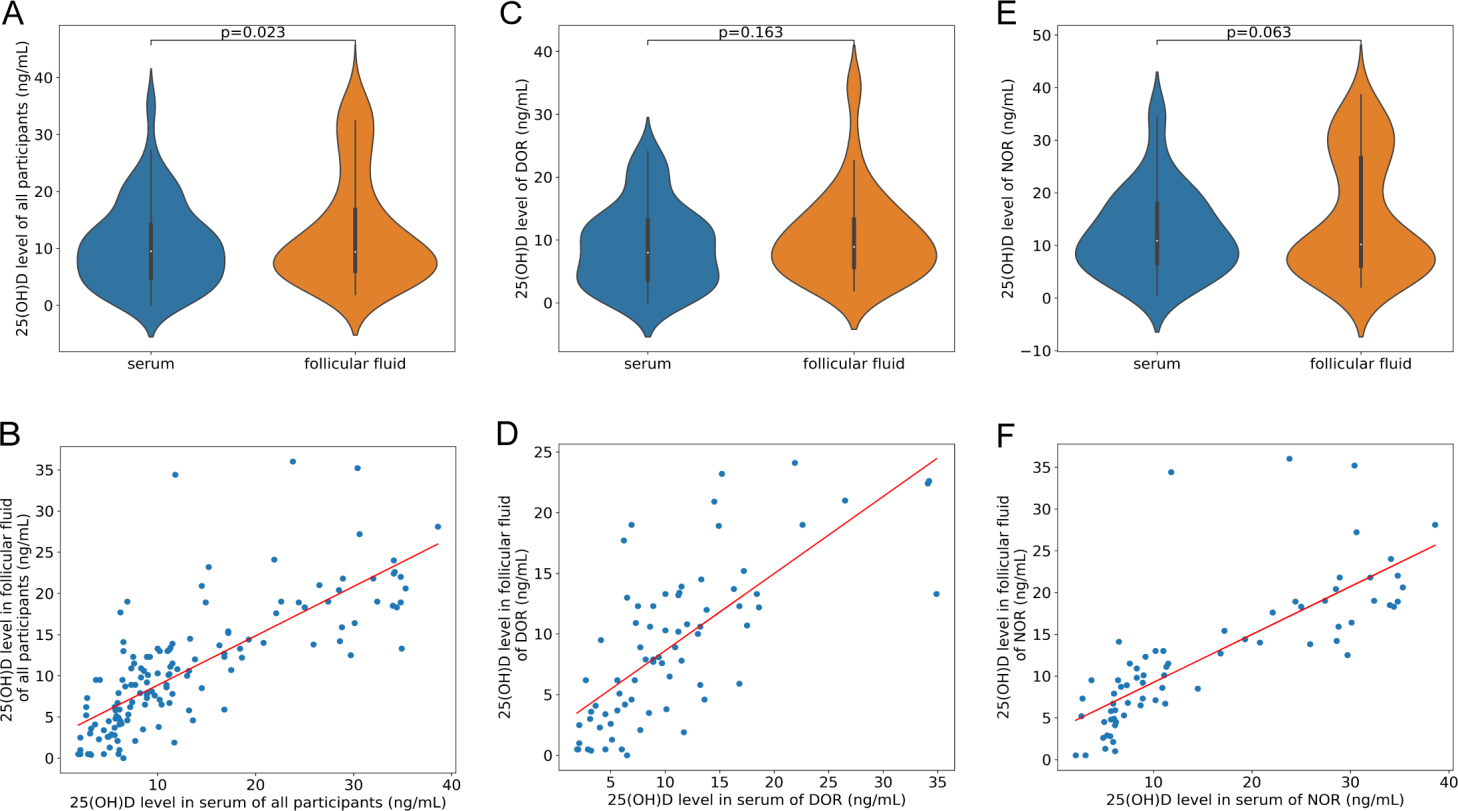
Violin-plot and correlation of serum and follicular fluid 25(OH)D level. **(A)** Violin-plot of serum and follicular fluid 25(OH)D level in all participants; **(B)** correlation of serum and follicular fluid 25(OH)D level in all participants (n= 145); **(C)** Violin-plot of serum and follicular fluid 25(OH)D level in DOR; **(D)** correlation of serum and follicular fluid 25(OH)D level in DOR (n = 74); **(E)** Violin-plot of serum and follicular fluid 25(OH)D level in NOR; **(F)** correlation of serum and follicular fluid 25(OH)D level in NOR (n = 71). Statistical significance for the distributional differences in the violin plots was assessed using the Mann-Whitney U test for independent group comparisons.

In this study, the 25(OH)D levels in serum and FF across all 145 participants demonstrated a high degree of consistency. In the total population, a strong and significant positive correlation was identified between VD-S and VD-FF levels (r = 0.769, P < 0.001, **Figure 1B**). Furthermore, violin plot analysis using the Mann-Whitney U test revealed a statistically significant difference between the concentration subgroups (P = 0.023, **Figure 1A**). In stratified analysis, this robust correlation was maintained in both the NOR group (r = 0.782, P < 0.001, **Figure 1D**) and the DOR group (r = 0.716, P < 0.001, **Figure 1F**). Notably, while the correlation remained stable, the distributional differences between serum and FF did not reach statistical significance when the subgroups were analyzed independently, with P = 0.163 for the NOR group (**Figure 1C**) and P = 0.063 for the DOR group (**Figure 1E**).

### 3.3 Exploratory subgroup analysis in patients with DOR based on 25(OH)D levels

To further investigate the impact of VD on patients with DOR, this group was stratified into VD non-deficient (VDH) and deficient (VDL) subgroups based on either VD-S or VD-FF levels.

When stratified by VD-S, the VDH subgroup (n=23) had significantly higher levels of both serum and follicular fluid VD compared to the VDL subgroup (n=51) (*P* < 0.001). Among metabolic markers, the VDH subgroup showed significantly higher HDL levels (1.60 *vs*. 1.36 mmol/L, *P* < 0.001) and significantly lower TSH levels (2.29 *vs*. 3.20 mIU/ml, *P* = 0.037). With respect to IVF outcomes, the oocyte retrieval rate (95.0% *vs*. 84.6%, *P* = 0.021) and the normal fertilization rate (72.9% *vs*. 56.3%, *P* = 0.016) were significantly higher in the VDH subgroup. No significant differences were found in age, BMI, basal sex hormones, AMH, AFC, other metabolic markers, or embryo development potential between the subgroups (*P* > 0.05) (**Table 2**).

**Table 2:** Exploratory subgroup analysis of clinical characteristics and IVF laboratory parameters based on serum 25 (OH)D levels in patients with diminished ovarian reserve.

Index	VDH (n=23)	VDL (n=51)	*Z/χ²*	*P*
Age (year)	34 [32-37]	36 [34-38]	-1.111	0.267
Duration of infertility (years)	3 [1-4]	3 [1-7]	-0.594	0.552
BMI	22.30 [20.90-24.60]	22.90 [20.70-25.70]	-0.169	0.865
bFSH (mIU/ml)	9.60 [7.50-12.00]	10.50 [7.73-15.40]	-0.853	0.394
bLH (mIU/ml)	5.07 [3.12-9.64]	5.87 [3.95-8.08]	-0.368	0.713
bE_2_ (pg/ml)	38.5 [22.30-57.70]	46.3 [20.20-62.60]	-0.023	0.981
P_4_ (ng/mL)_)_	0.3 [0.17-0.59]	0.24 [0.15-0.32]	-1.741	0.085
PRL (ng/mL)	16.99 [13.60-26.40]	18.9 [15.1-26.8]	-0.929	0.353
AMH (ng/mL)	0.6 [0.21-0.85]	0.42 [0.24-0.69]	-0.958	0.338
TSH (mIU/ml)	2.29 [1.58-3.38]	3.2 [2.26-4.62]	-2.085	0.037
AFC	5 [4-6]	4 [3-6]	-1.501	0.133
HCY (μmol/L)	10.4 [8.90-15.00]	10.7 [8.6-15]	-0.175	0.861
FBG (mmol/L)	4.63 [4.50-5.40]	4.8 [4.50-5.18]	-0.082	0.935
TG (mmol/L)	1.27 [0.91-1.63]	1.23 [0.87-1.84]	-0.076	0.939
TC (mmol/L)	4.46 [3.40-5.03]	4.37 [3.93-5]	-0.310	0.757
HDL (mmol/L)	1.60 [1.47-1.99]	1.36 [1.17-1.57]	-3.418	<0.001
LDL (mmol/L)	2.82 [2.43-3.33]	2.69 [2.40-3.20]	-0.450	0.653
ESR (mm/h)	5 [2.0-7.0]	6 [4.0-8.8]	-1.292	0.196
VD-S (ng/mL)	16.80 [13.60-21.90]	6.90 [4.10-9.40]	-6.851	<0.001
VD-FF (ng/mL)	13.30 [10.70-20.90]	6.20 [2.03-10.20]	-4.907	<0.001
Oocytes retrieval rate (%)	95.0% (95/101)	84.6% (126/149)	5.293	0.021
Metaphase II oocytes rate (%)	89.5% (85/95)	88.9% (112/126)	0.019	0.890
Normal fertilization rate (%)	72.9% (62/85)	56.3% (63/112)	5.805	0.016
Available embryos rate of D3 (%)	64.5% (40/62)	65.1% (41/63)	0.004	0.947
High quality embryos rate of D3 (%)	27.4% (17/62)	23.8% (15/63)	0.214	0.644

25 (OH)D levels are presented in ng/mL. To convert to nmol/L, multiply the values by 2.5 (e.g., 12 ng/mL) = 30 nmol/L.

FSH, basal follicle-stimulating hormone; LH, basal luteinizing hormone; E2, estradiol; P4, progesterone; PRL, prolactin; AMH, anti-Mullerian hormone; TSH, thyroid-stimulating hormone; bAFC, basal antral follicle count; FBG, fasting blood glucose; TG,triglycerides; TC, cholesterol; HDL, high-density lipoprotein; LDL, low-density lipoprotein; VD-S, 25 (OH)D level in serum; VD-FF, 25 (OH)D level in follicular fluid.

All comparisons in this table are exploratory. The study was not independently powered for these specific subgroup analyses.

Similar findings were observed when patients were stratified by VD-FF levels. The VDH subgroup (n=25) had significantly higher HDL levels (1.57 *vs*. 1.36 mmol/L, *P* = 0.017) and significantly lower ESR levels (4 *vs*. 7 mm/h, *P* = 0.024) compared to the VDL subgroup (n=49). Likewise, the oocyte retrieval rate (95.8% *vs*. 83.9%, *P* = 0.004) and the normal fertilization rate (75.0% *vs*. 54.9%, *P* = 0.004) were significantly superior in the VDH subgroup. No significant differences were noted for other baseline clinical characteristics or embryological parameters between these subgroups (*P* > 0.05) (**Table 3**).

**Table 3:** Exploratory subgroup analysis of clinical characteristics and IVF laboratory parameters based on follicular fluid 25 (OH)D levels in patients with diminished ovarian reserve.

Index	VDH (n=25)	VDL (n=49)	*Z/χ²*	*P*
Age (year)	34 [32.5-37]	37 [34-38]	-1.500	0.134
Duration of infertility (years)	3 [2-4.5]	3 [1-7]	-0.294	0.769
BMI	21.7 [20.85-23.72]	23.4 [20.70-25.90]	-1.698	0.090
bFSH (mIU/ml)	10.1 [7.72-15.00]	10.9 [7.54-15.05]	-0.274	0.784
bLH (mIU/ml)	5.87 [3.77-8.76]	5.6 [3.17-8.86]	-0.029	0.977
bE_2_ (pg/ml)	41.2 [24.5-74.6]	43.7 [20.20-58.90]	-0.651	0.515
P_4_ (ng/mL)_)_	0.26 [0.18-0.41]	0.24 [0.16-0.32]	-1.161	0.246
PRL (ng/mL)	18 [15.00-23.50]	18.9 [13.90-26.65]	-0.411	0.681
AMH (ng/mL)	0.53 [0.21-0.74]	0.46 [0.25-0.72]	-0.309	0.758
TSH (mIU/ml)	2.75 [1.70-4.63]	3.03 [1.86-4.44]	-0.606	0.545
AFC	4 [4-6]	4 [3-6]	-1.231	0.218
HCY (μmol/L)	10.7 [7.55-14.05]	10.6 [9.05-16.45]	-1.000	0.317
FBG (mmol/L)	4.63 [4.50-5.27]	4.85 [4.52-5.21]	-0.846	0.398
TG (mmol/L)	1.27 [0.94-1.85]	1.19 [0.79-1.78]	-0.634	0.526
TC (mmol/L)	4.18 [3.47-4.92]	4.46 [3.99-5.27]	-1.434	0.151
HDL (mmol/L)	1.57 [1.43-1.86]	1.36 [1.18-1.62]	-2.378	0.017
LDL (mmol/L)	2.60 [2.22-3.02]	2.74 [2.48-3.59]	-1.863	0.062
ESR (mm/h)	4 [2-7]	7 [4-10]	-2.259	0.024
VD-S (ng/mL)	14.09 [10.60-20.25]	7.20 [4.05-10.25]	-4.989	<0.001
VD-FF (ng/mL)	13.90 [13.10-19.95]	4.60 [2.30-8.00]	-7.002	<0.001
Oocytes retrieval rate (%)	95.8% (91/95)	83.9% (130/155)	8.159	0.004
Metaphase II oocytes rate (%)	92.3% (84/91)	86.9% (113/130)	1.603	0.205
Normal fertilization rate (%)	75.0% (63/84)	54.9% (62/113)	8.421	0.004
Available embryos rate of D3 (%)	66.7% (42/63)	62.9% (39/62)	0.194	0.660
High quality embryos rate of D3 (%)	27.0% (17/63)	24.2% (15/62)	0.128	0.721

25 (OH)D levels are presented in ng/mL. To convert to nmol/L, multiply the values by 2.5 (e.g., 12 ng/mL) = 30 nmol/L.

FSH, basal follicle-stimulating hormone; LH, basal luteinizing hormone; E2, estradiol; P4, progesterone; PRL, prolactin; AMH, anti-Mullerian hormone; TSH, thyroid-stimulating hormone; bAFC, basal antral follicle count; FBG, fasting blood glucose; TG, triglycerides; TC, cholesterol; HDL, high-density lipoprotein; LDL, low-density lipoprotein; VD-S, 25 (OH)D level in serum; VD-FF, 25 (OH)D level in follicular fluid.

All comparisons in this table are exploratory. The study was not independently powered for these specific subgroup analyses.

## 4 Discussion

Diminished ovarian reserve (DOR) is a key challenge in IVF. Our core finding shows that women with DOR have significantly lower VD-S and VD-FF levels than controls. Within the DOR group, those with higher VD levels had significantly better oocyte retrieval and fertilization rates, alongside higher HDL cholesterol. These results suggest that VD deficiency is linked to suboptimal IVF laboratory parameters in DOR patients, potentially reflecting an association with oocyte quality and lipid metabolism.

The dual analysis of violin and scatter plots (**Figure 1**) elucidates the complex relationship between systemic and local 25(OH)D levels. The strong correlation coefficients (r > 0.7) indicate that peripheral VD status is the primary driver of the follicular microenvironment. However, the significant difference observed in the violin plot for the total population (P = 0.023) suggests the existence of a local metabolic gradient or a selective sequestering mechanism within the follicle. Interestingly, the marginal difference observed in the DOR subgroup (P = 0.063), contrasted with the NOR group (P = 0.163), may hint at subtle alterations in the efficiency of VD transport or local utilization in patients with diminished ovarian reserve.

One key finding of this study is that VD-S levels were significantly lower in the DOR group compared with controls, supporting the hypothesis that VD deficiency may be a risk factor for diminished ovarian reserve. Consistently, animal studies have shown reduced VD levels in models of premature ovarian failure (19). Mechanistically, VD may regulate ovarian function partly through its effect on AMH, as the AMH gene promoter contains a VD response element that enables VDR-mediated up regulation of AMH expression (20, 21). However, despite lower AMH levels in the DOR group than in the NOR group, no significant difference was observed between high- and low-VD subgroups within DOR. Similar findings from cross-sectional studies suggest that VD-S and AMH are not linearly correlated (22, 23). This may indicate that chronic VD deficiency is involved in the pathophysiology of DOR, whereas transient changes in VD may not immediately influence AMH levels, implying a cumulative rather than instantaneous effect.

The core finding of this study is that in the specific population of women with diminished ovarian reserve (DOR), non-deficient VD levels are significantly associated with improved oocyte retrieval and normal fertilization rates. This result provides new evidence for the role of VD in female reproduction, particularly in patient populations facing challenges with ovarian function. Our findings are consistent with a prospective study that also focused on DOR patients, which confirmed that higher 25(OH)D levels in follicular fluid led to a greater number of retrieved oocytes, mature oocytes, and higher normal fertilization rates (24). Additionally, another study in a general infertile population found that individuals with sufficient follicular fluid VD levels had more normally fertilized oocytes and high-quality embryos (25). However, the association between VD levels and assisted reproductive technology (ART) outcomes remains a subject of debate in the academic community. Some studies have not observed a significant association between VD levels and embryo quality or pregnancy outcomes (26, 27). These discrepancies in conclusions may arise from the heterogeneity of study populations (e.g., physiological differences between DOR and normal ovarian reserve populations), different diagnostic criteria for VD deficiency, and variations in study design and potential confounding factors. Notably, this study adopted a threshold of 12 ng/mL (30 nmol/L) to define VD deficiency, adhering to the Expert Consensus on the Evaluation and Improvement of VD Nutritional Status in China (2023). While this cutoff is specifically tailored to the Chinese population, it is lower than the 20 ng/mL or 30 ng/mL thresholds frequently utilized in international reproductive literature. This relatively conservative definition may influence the comparability of our findings with studies conducted in Western cohorts. Specifically, our VDH group might encompass individuals who would be classified as “insufficient” by international standards, potentially diluting the observed effect size. Conversely, our VDL group identifies a population with more severe VD depletion. Therefore, while our results are highly relevant to clinical practice in China, their generalizability to populations with different baseline VD levels should be interpreted with caution.

This study confirms a high correlation between VD-S and VD-FF levels, which aligns with previous findings (25, 28) and implies that VD-S can serve as a reliable indicator for assessing the microenvironment surrounding the oocyte. The follicular fluid is the immediate environment for oocyte development and maturation, where VD is hypothesized to be related to oocyte quality, potentially through its role in folliculogenesis and steroidogenesis (29, 30). While this study focused on total 25(OH)D as a primary clinical marker, it is important to consider the complexity of the VD metabolic spectrum. The “Free Hormone Hypothesis” suggests that the biological activity of vitamin D may be more accurately reflected by its unbound, free form rather than the total concentration, which is largely sequestered by Vitamin D-binding protein (VDBP) (31). Recent studies utilizing liquid chromatography-tandem mass spectrometry (LC-MS/MS) to measure the full range of VD metabolites in follicular fluid have indicated that while total 25(OH)D is highly correlated with its metabolites, the bioavailability regulated by VDBP plays a critical role in follicular steroidogenesis and oocyte competency (8). By identifying a significant association between lower total 25(OH)D and reduced fertilization rates, our findings provide a necessary clinical foundation that aligns with these more granular metabolic investigations. We hypothesize that lower systemic VD levels lead to a depleted follicular pool of bioavailable VD, which in turn impairs the antioxidant capacity of the follicular microenvironment. We observed that VD levels primarily affected the early stage of fertilization, with no significant impact on the developmental potential of day-3 embryos. We speculate that the key role of VD may be concentrated on oocyte final maturation and fertilization competence. Although some research indicates that higher follicular fluid VD levels are associated with a greater proportion of high-quality embryos in DOR patients (24) and that VD supplementation can improve embryo quality (32), this effect may not be universal. A possible explanation is that once fertilization is successful, the embryo’s own genetic potential and the *in vitro* culture conditions become the dominant factors driving its subsequent development. While the foundation of a high-quality oocyte established by VD is crucial, its influence may diminish in the later stages.

Notably, in the diminished ovarian reserve (DOR) patient cohort, the VD non-deficient subgroup exhibited significantly higher high-density lipoprotein cholesterol (HDL) levels. This observation aligns with findings in other populations; for instance, lower VD levels are associated with lower HDL levels in postmenopausal women (33). Furthermore, a positive association between VD status and HDL has also been noted in individuals with kidney stone disease and Hashimoto’s thyroiditis (34). VD is recognized as a beneficial compound for managing abnormal lipid metabolism in postmenopausal women (35) and is known to influence lipoprotein function and metabolism through various pathways (36).

Based on these findings, we propose a “metabolic-inflammation axis” hypothesis to explain the potential mechanism of VD’s impact on IVF outcomes. It is known that dysregulation of lipid metabolism is closely linked to a pro-inflammatory state (35), and VD itself possesses potent immunomodulatory functions, capable of reducing systemic inflammatory mediators and promoting the release of anti-inflammatory cytokines (37, 38). Therefore, a plausible mechanism is that VD status is potentially associated with IVF parameters through its link to lipid metabolism, particularly by increasing levels of anti-inflammatory and antioxidant HDL. This, in turn, could mitigate the systemic or local ovarian micro-inflammatory state that is detrimental to oocyte development, ultimately optimizing oocyte quality. This hypothesis connects the metabolic improvements observed in our study with the enhanced IVF laboratory parameters, offering an integrated perspective on the role of VD in reproductive health.

The subgroup analysis in our study also revealed lower levels of thyroid-stimulating hormone (TSH) and erythrocyte sedimentation rate (ESR) in the VD non-deficient group, which further supports the systemic regulatory role of VD. The finding of lower TSH is consistent with a growing body of evidence, as multiple studies have confirmed that VD supplementation can decrease TSH levels in patients with subclinical hypothyroidism and autoimmune thyroiditis (39, 40). Moreover, a negative correlation between VD levels and TSH has been frequently reported across various populations (41–43). Concurrently, as a non-specific marker of inflammation, the reduction in ESR aligns with the established anti-inflammatory properties of VD, which has been shown to suppress the production of pro-inflammatory cytokines (36). Taken together, these findings suggest that VD status may be associated with a more favorable systemic environment for successful pregnancy by concurrently modulating thyroid function and inflammation levels.

The primary strength of this study lies in its prospective design and the concurrent analysis of serum and follicular fluid, which enables a direct investigation of the follicular microenvironment in the clinically challenging DOR population. Notably, strict seasonal control was achieved by restricting recruitment to winter months, thereby capturing the nadir of endogenous vitamin D synthesis and minimizing the confounding effects of sunlight exposure. Furthermore, by focusing on laboratory metrics (oocyte retrieval and fertilization rates), this study provides a proximal and direct assessment of oocyte competency, minimizing the impact of downstream confounders such as endometrial receptivity. These findings serve as a critical scientific foundation to justify future randomized controlled trials (RCTs).

Several limitations must be acknowledged. First, due to the single-center design and limited sample size, the subgroup analyses (VDH *vs*. VDL) were not independently powered and should be considered exploratory and hypothesis-generating. Second, our metabolic assessment was limited to total 25(OH)D measured via automated immunoassay, which reflects standard clinical practice but lacks the precision of LC-MS/MS. The absence of data on Vitamin D Binding Protein (VDBP) and free 25(OH)D restricts our ability to mechanistically elucidate the role of bioavailable vitamin D. Finally, the use of a 12 ng/mL cutoff, while aligned with the Chinese expert consensus, along with the lack of long-term clinical outcomes (e.g., live birth rates), may limit the generalizability of our findings to populations using international thresholds.

## 5 Conclusion

In conclusion, this study demonstrates a significant positive association between VD levels in both serum and follicular fluid and key IVF laboratory outcomes—specifically, oocyte retrieval and normal fertilization rates—in patients with diminished ovarian reserve. Based on these results, routine screening of VD-S may be considered in patients with DOR may be considered to assess their VD status. For those with VD deficiency, standardized supplementation before an IVF cycle could be considered as a potential, economical, and safe adjuvant strategy, although its clinical efficacy remains to be validated through further interventional research. Looking forward, there is an urgent need for large-scale, multicenter randomized controlled trials (RCTs) to confirm the effect of VD supplementation on live birth rates in DOR patients. Future studies should also explore the molecular mechanisms by which VD influences oocyte quality and aim to define the optimal dosage and timing for supplementation to provide precise clinical guidance.

## Data Availability

The original contributions presented in the study are included in the article/supplementary material. Further inquiries can be directed to the corresponding author.
